# UAV-Based Oil Leakage Spot Detection Under Complex Illumination via a Collaborative Low-Light Enhancement and Detection Framework

**DOI:** 10.3390/s26061819

**Published:** 2026-03-13

**Authors:** Yunsheng Ha, Ling Zhao, Huili Zhang

**Affiliations:** 1 College of Management and Economics, Tianjin University, Tianjin 300072, China; 2 College of Computer and Information Technology, Northeast Petroleum University, Daqing 163318, China

**Keywords:** UAV inspection, oil leakage spot detection, low-light image enhancement, Retinex, YOLOv11, attention mechanism

## Abstract

Accurate detection of oil leakage spots is essential for oilfield safety and environmental protection. However, UAV-based inspection in onshore oilfields often suffers from complex illumination conditions, such as low light, backlighting, and mixed shadows, which simultaneously degrade image visibility and obscure leakage-sensitive features, thereby causing missed detection of minute and weak-texture oil leakage targets. Unlike generic low-light enhancement or object detection tasks, the core challenge of onshore UAV oil leakage inspection lies in preserving leakage-oriented fine cues during enhancement while improving the detector’s ability to distinguish leakage targets from highly confusing oilfield backgrounds. To address this task-specific challenge, we propose a collaborative low-light enhancement and detection framework that jointly optimizes leakage-detail-preserving enhancement and multi-scale interference-suppressed detection. Specifically, an improved Retinex-based enhancement network is designed by integrating multi-scale feature aggregation, NAFNet-based denoising, and a CBAM attention mechanism to enhance brightness while preserving leakage details. The enhanced images are then fed into an improved YOLOv11 detector, where an AC-FPN module is adopted to strengthen multi-scale feature fusion and suppress background interference. Experiments on UAV oilfield datasets demonstrate that the proposed method achieves a precision of 94.25% and a mean average precision (mAP) of 87.54%, outperforming existing approaches. The proposed framework provides an effective and robust solution for oil leakage spot detection under complex illumination.

## 1. Introduction

Oilfield production operations are a high-risk industry. Crude oil possesses toxic and hazardous properties, and under high-temperature and high-pressure conditions, leaks can easily trigger explosions, posing a serious threat to oil and gas production safety and the ecological environment. Therefore, developing efficient and precise oil spill monitoring technology is of paramount importance [1]. In recent years, drone inspections have become the mainstream technology for oilfield leak prevention and control due to their advantages of wide coverage, high mobility, and non-contact detection. However, in complex field environments, they often encounter adverse lighting conditions such as low light, backlighting, and intersecting light and shadow. This leads to image quality defects like detail loss and increased noise, directly causing confusion between leak targets and background features. This is particularly prone to causing missed detections of minute oil film leaks, severely limiting detection accuracy and robustness [2]. Meanwhile, existing research on oil leakage detection in oilfields exhibits significant shortcomings: foreign studies predominantly focus on marine oil spill detection, such as Calla et al.’s use of satellite multi-frequency microwave sensors to measure the extent and thickness of surface oil spills [3], Research on oil spill detection for onshore oilfields remains relatively scarce. While domestic studies have recognized the importance of remote video monitoring for oilfield production management—which can enhance production efficiency and safety reliability [4], However, existing monitoring systems largely rely on sensors such as pressure transmitters and flow meters to collect equipment parameters [5], The system exhibits limited capability for direct visual detection of oil seepage targets and lacks optimization for image quality under complex outdoor lighting conditions, making it difficult to meet the precise detection requirements in the complex scenarios of onshore oilfields [6].

Low-light image enhancement (LLIE) is a key technology in the field of computer vision [7]. Its core objective is to enhance image visibility and contrast in low-light scenarios, effectively improving image quality under conditions such as uneven illumination [8], extreme darkness [9], and backlighting [10]. High-quality images significantly boost the performance of advanced visual tasks like image recognition and object detection [11], providing essential technical support for addressing the complex lighting challenges inherent in drone inspections. Reviewing research progress in the LLIE field, early methods primarily focused on distribution mapping [12] and model optimization [13], but commonly suffered from issues such as noise amplification, color distortion, uneven exposure, and computational complexity. In recent years, deep learning-driven LLIE methods have achieved breakthrough progress. Techniques such as LLNet [14], Zero-DCE [15], and LEGAN [16] have significantly enhanced enhancement effects, though some methods suffer from artifacts and insufficient generalization. Among these, RetinexNet—a classic deep learning framework integrating brightness enhancement and noise reduction—effectively boosts image brightness by leveraging Retinex theory aligned with human visual perception. However, it exhibits notable limitations in oilfield image enhancement: the decomposition stage tends to lose fine-grained details like oil spill edges and textures; reflection component denoising is incomplete; and insufficient detail preservation during brightness enhancement directly leads to false negatives and false positives in subsequent oil spill detection [17].

It should be emphasized that the problem addressed in this study is not a generic low-light object detection task, but a UAV-based oil leakage spot detection task for onshore oilfield inspection scenarios. Compared with general low-light detection problems, oil leakage detection in oilfields has stronger task-specific characteristics. First, early-stage leakage targets often appear as minute oil films, strip-like seepage traces, or irregular diffuse patches, which are small in scale, weak in boundary response, and limited in discriminative texture. Second, leakage regions are easily confused with soil, vegetation shadows, stains on equipment surfaces, and specular reflections from wet areas because they share similar color, brightness, and texture patterns. Third, the reflective, absorptive, and local highlight properties of crude oil vary significantly with illumination direction, viewing angle, and ground material, making the same leakage target visually unstable under low light, backlighting, and mixed shadow conditions. Fourth, oilfield safety supervision requires timely identification of tiny early-stage leaks, which means that the enhancement stage should not only improve visibility but also preserve leakage-sensitive cues such as edges, textures, and scale information. Therefore, the core challenge of this task is not merely to detect objects under poor illumination, but to achieve leakage-oriented detail-preserving enhancement and accurate detection under complex illumination interference.

Beyond the algorithmic perspective, UAV-based inspection also offers important workflow advantages for onshore oilfield monitoring. Similar UAV-imagery inspection pipelines have been reported in other domains such as construction-site rebar counting, where labeled UAV datasets and augmentation-based detection studies were developed to support remote visual inspection tasks. These studies illustrate that UAV platforms can efficiently acquire overhead imagery for object-centric inspection and provide structured visual data for downstream computer-vision analysis [18]. In practical inspection scenarios, UAVs are attractive because they can rapidly cover large areas, reach hazardous or hard-to-access locations, reduce the need for manual patrols at height or in unsafe zones, and improve personnel safety while generating reviewable image records. However, UAV deployment also involves practical trade-offs. Flight missions are constrained by battery endurance, weather and wind sensitivity, possible occlusions from surrounding structures or vegetation, and the need for stable viewpoints and trained operators. In addition, real deployments must comply with site-specific privacy requirements and applicable airspace regulations, including drone registration, remote-pilot certification, and airspace authorization when required. Therefore, the proposed method should be viewed not only as a vision algorithm, but also as a perception module within a broader UAV inspection workflow [19].

In summary, the problem addressed in this paper is defined as follows: in UAV-based onshore oilfield inspection scenarios, how can minute, weak-texture, irregularly shaped, and background-confusing oil leakage targets be accurately detected under complex illumination conditions such as low light, backlighting, and mixed shadow? This problem is different from generic object detection tasks. Its difficulty lies not only in insufficient brightness, but also in the fact that complex illumination simultaneously weakens leakage-related edges and textures, amplifies background interference, and increases the instability of recognizing leakage targets at different scales. Although existing general LLIE methods can improve image visibility, they lack leakage-aware constraints for preserving discriminative cues; meanwhile, generic detectors are still limited in distinguishing leakage targets from oilfield background clutter in enhanced images. Therefore, the crux of this work is not simply to combine image enhancement and object detection, but to build a task-oriented collaborative framework in which enhancement is constrained by leakage-detail preservation and detection is strengthened by illumination-robust multi-scale feature representation. The novelty of this study lies in tailoring the entire “enhancement–detection” pipeline to the visual characteristics of onshore UAV oil leakage scenarios, where minute leakage targets, weak textures, irregular shapes, and highly confusing backgrounds must be handled jointly under complex illumination.

## 2. Method Overview

**Figure 1:**
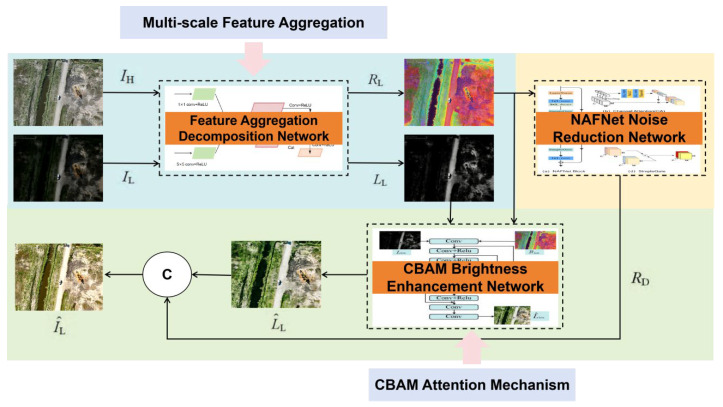
Schematic diagram of the improved low-light image enhancement network architecture.

To address the challenges of low accuracy and missed detection of minute targets in drone oil leakage identification under complex lighting conditions, while simultaneously resolving issues of detail loss and noise interference in low-light images, this paper designs an experimental workflow centered on two core components: low-light enhancement and oil leakage detection. Retinex theory posits that observed images can be decomposed into the product of reflection and illumination components. Inspired by this, we constructed an end-to-end deep neural network architecture, instantiating the theoretical decomposition, denoising, and enhancement processes as learnable neural network modules. The eNAFNet andstage employs an improved RetinexNet approach (optimizing the decomposition network via a feature fusion module, constructing the denoising network with NAFNet, and enhancing the brightness enhancement network using Unet + CBAM (Channel-Spatial Dual Attention)) to improve image quality. The detection training stage utilizes an improved YOLOv11 framework [20] (replacing the original FPN module with AC-FPN: contextual enhancement module + attention-guided module (CxAM + CnAM)) for precise detection. The architecture of the improved low-light image enhancement network is illustrated in Figure 1.

As shown in Figure 1, during the training phase, both normally lit oilfield images (*I*_H_) and low-light oilfield images (*I*_L_) captured by the UAV are input into the network simultaneously. However, during the testing phase, only low-light oilfield images captured by the UAV are accepted as input. The entire network primarily consists of three core components: ① Decomposition module based on multi-scale feature aggregation: simultaneously inputting normal/low-light oilfield image pairs captured by UAVs, the decomposition network separately extracts the illumination component *L*_L_ (carrying image brightness information) and the reflectance component *R*_L_ (containing core features such as oil spill edges and textures) as inputs for subsequent modules; the improved decomposition network incorporates a multi-scale feature aggregation module to capture shallow-level details across different receptive fields (e.g., textures of minute oil film leaks, spectral characteristics of oil spills across different bands), thereby generating high-quality *L*_L_ and *R*_L_ components that lay the foundation for preserving oil spill features in subsequent processing. ② NAFNet-based noise reduction module: It should be emphasized that the input of NAFNet is not the output of any single-scale branch, but the aggregated and projected reflectance component RL. Therefore, the front-end 1 × 1, 3 × 3, and 5 × 5 convolutions jointly construct a reflectance representation suitable for denoising and detail preservation from the perspectives of channel contrast, local structure, and broader contextual cues. Receives the *R*_L_ component from the decomposition network’s low-light oilfield image. Targeting spectral noise present in the *R*_L_ component under drone low-light conditions, NAFNet performs hierarchical processing of *R*_L_’s noise features to achieve precise noise suppression, reconstructing a purer *R*_D_ component. ③ Brightness enhancement module based on CBAM optimization: Receives the *L*_L_ component from the decomposition network. Utilizes a U-Net architecture with skip connections incorporating CBAM attention mechanisms. Performs brightness enhancement on *L*_L_ under the constraint of reflectance component features—CBAM adaptively focuses on channels and spatial regions critical for oil spill detection, addressing detail loss in low-light conditions. Outputs the enhanced luminance component $\overset{\wedge }{L}$_L_. Finally, multiplying the enhanced illumination map $\overset{\wedge }{L}$_L_ from the brightness enhancement module with the reduced noise reflection map *R*_D_ yields the enhanced UAV oilfield image $\overset{\wedge }{I}$_L_. This image fully preserves multiscale details and features of oil spills, providing high-quality data input support for subsequent oil spill detection tasks.

**Figure 2:**
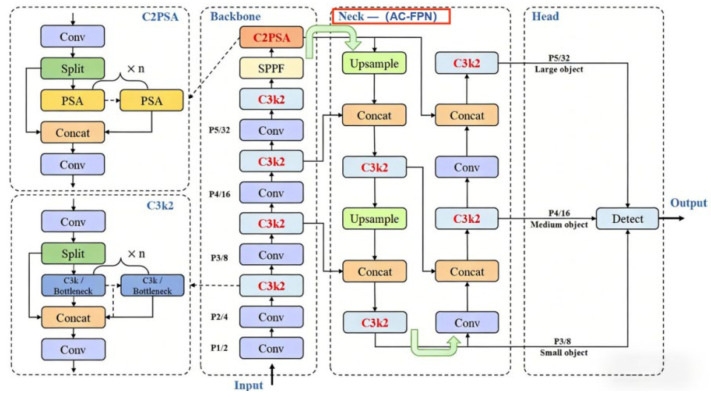
Schematic diagram of the improved yolov11 network architecture.

YOLOv11, as the latest iteration of the YOLO series object detection models [21], achieves significant breakthroughs in architectural design, feature representation capabilities, and edge computing efficiency. Its innovative integration of C3k2 blocks, SPPF components, and C2PSA modules efficiently adapts to the multi-scale feature extraction and complex background discrimination demands of drone oil leak detection scenarios. This model follows the classic three-stage structure comprising a backbone network (feature extraction), neck network (feature fusion), and detection head (classification and localization), where the original neck network employs a feature pyramid network (FPN). To fully extract oil leak target features from augmented data and achieve precise detection in complex lighting conditions for UAV scenarios, this paper designs the YOLOv11-AC-FPN oil leak detection network architecture. Its core improvement strategy involves replacing the traditional feature pyramid network (FPN) with the AC-FPN module within the native YOLOv11 architecture, while preserving the rest of the architecture to maintain the model’s lightweight and low computational consumption advantages. This adaptation addresses the engineering requirements for real-time drone-based inspections. The improved YOLOv11 network architecture is illustrated in Figure 2.

Specifically, the left encoding side functions as a multi-scale feature extraction unit. Taking the enhanced dataset as input, it extracts features layer by layer through the synergistic interaction between the CBS component and the DWC layer. This enables precise capture of both the shallow texture features of minute oil film leaks in low-altitude drone inspection scenarios and the deep structural features of large-area diffuse oil films in high-altitude inspection scenarios, complementing the feature advantages of the enhanced data. The AC-FPN in the central fusion region serves as the core performance enhancement module. Its embedded contextual enhancement module (CEM) effectively integrates feature information across different levels, strengthening contextual associations for multi-scale oil spill targets. This overcomes the technical bottleneck of traditional FPNs’ inadequate adaptability to multi-scale oil spill targets. The attention module (AM) employs dual-dimensional coordination between the contextual attention submodule (CxAM) and content attention submodule (CnAM) to precisely capture semantic associations and spatial dependencies within feature maps. This effectively suppresses redundant interference from background elements like vegetation and drilling equipment in oilfield scenarios, further focusing on core oil spill features. Finally, the detection head module on the right receives the optimized feature maps output by AC-FPN to complete the classification and localization tasks for oil spills at different scales. Leveraging the high-quality feature representations from the augmented dataset and AC-FPN’s precise feature fusion capabilities, this network architecture achieves high-precision identification of oil spills in oilfield scenarios under unmanned aerial vehicle operations, providing reliable technical support for intelligent oilfield inspection.

## 3. Experimental Methods

### 3.1. Image Enhancement Algorithm Optimization

#### 3.1.1. Decomposition Network Based on Multi-Scale Feature Aggregation Module

**Figure 3:**
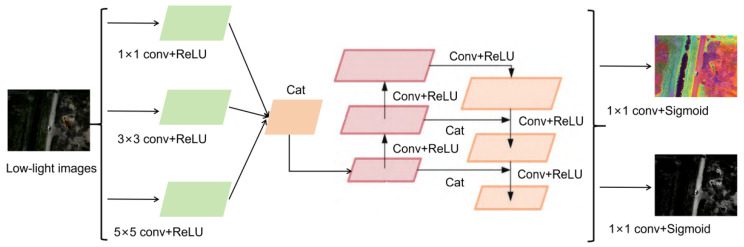
Image decomposition network architecture supported by multi-scale feature aggregation module.

To address the issues of insufficient capture of multi-scale oil spill features and loss of detail during the image decomposition process for low-light drone oilfield imagery, a multi-scale feature aggregation module was incorporated into the original fully convolutional neural network [22]. This module transmits both shallow and deep features through lateral connections to the deep network simultaneously, enabling the decomposition of higher-quality reflection component R and illumination component L. The improved decomposition network architecture is illustrated in Figure 3.

Figure 3 illustrates the decomposition network for low-light UAV oilfield images supported by the multi-scale feature aggregation module. Its main purpose is to achieve accurate separation of the reflectance and illumination components, thereby providing high-quality component representations for subsequent oil leakage detection. Considering that oil leakage targets under UAV observation simultaneously exhibit minute oil-film textures, irregular local boundaries, and coarse large-area diffuse distributions, this study adopts parallel convolution branches with kernel sizes of 1 × 1, 3 × 3, and 5 × 5 at the front end of the decomposition network to balance receptive-field coverage and computational cost.

Specifically, the 1 × 1 convolution branch is mainly used for inter-channel information recombination and local response recalibration. It enhances pixel-level intensity differences, gray/color contrast between oil films and background, and fine-grained reflectance responses, thereby providing a more stable low-level representation for subsequent reflectance decomposition. The 3 × 3 convolution branch is used to extract local neighborhood structures of leakage regions, with emphasis on minute leakage edges, strip-like seepage traces, and local morphological variations in medium-scale oil films. The 5 × 5 convolution branch expands the receptive field with relatively low parameter overhead, so as to capture broader contextual relationships and coarse contour cues, improving the modeling of the spatial continuity of large-area diffuse oil films and their relationships with surrounding shadows, vegetation, and ground reflections. It should be noted that the 5 × 5 branch is not intended to independently accomplish full “global contour” modeling; rather, it provides a larger-receptive-field prior, which is further fused with the 1 × 1 and 3 × 3 branches through channel concatenation and subsequent Conv + ReLU-based feature refinement, thereby forming a larger effective receptive field and enhancing the overall representation of multi-scale leakage features.

After channel-wise aggregation, the fused features are fed into the feature refinement path, where multiple convolution and activation operations perform hierarchical refinement and cross-layer propagation, further preserving fine-grained multi-scale information. Finally, the refined aggregated features are projected into the reflectance component RL and illumination component LL through two independent 1 × 1 convolution layers, followed by sigmoid constraints to the range of. The subsequent NAFNet module takes the aggregated and projected reflectance component RL, rather than the raw output of any single branch, as its input. Therefore, the channel recalibration and low-level contrast cues provided by the 1 × 1 branch are complementary to the structural and contextual information extracted by the 3 × 3 and 5 × 5 branches, jointly providing a more suitable input basis for noise suppression and detail preservation in NAFNet.

#### 3.1.2. Noise-Aided Filtering Network Based on NAFNet

**Figure 4:**
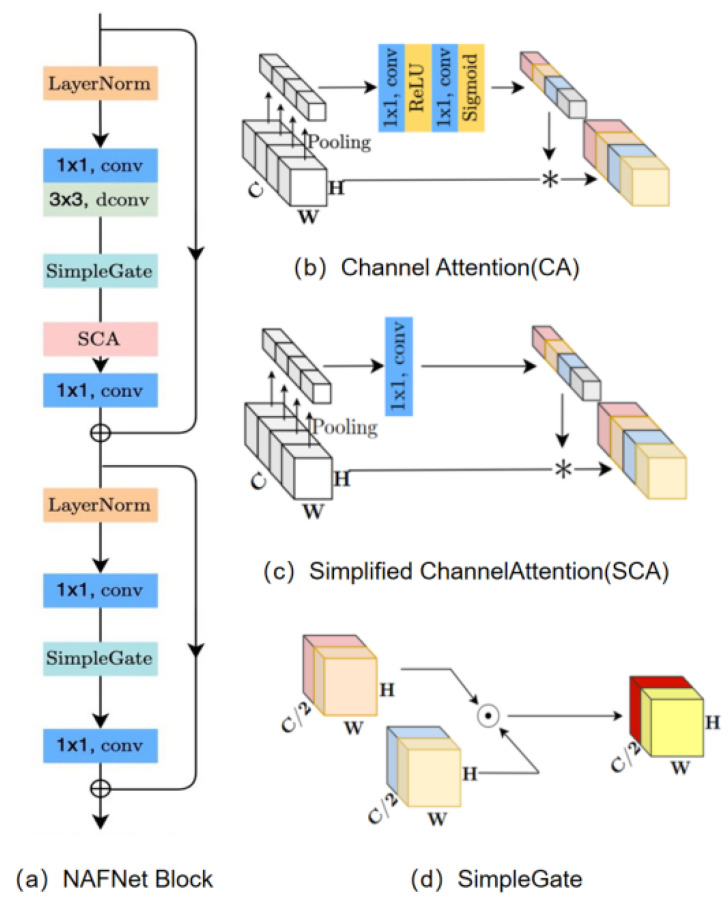
Noise Reduction Network Model.

To address the issue of poor image enhancement caused by noise, a noise reduction network based on NAFNet [23] was designed. Its overall structure is shown in Figure 4, where ☉ denotes matrix dot product and * denotes channel-wise multiplication.

In Figure 4, the denoising module employs UNet as its network architecture, utilizing a block-based skip-connection stacking approach to significantly reduce complexity between internal blocks and minimize image detail loss during denoising. Within the NAFNetBlock structure, noise of varying intensities undergoes LN normalization processing, enabling smoother training. The SimpleGate activation function replaces nonlinear activations to simplify the model structure. The introduction of spatial and channel attention (SCA) balances computational efficiency with the incorporation of global information while suppressing irrelevant features. NAFNet fully leverages effective features to preserve detail during denoising. Finally, the reflected component decomposed by the network undergoes processing through the denoising network to yield a noise-suppressed reflected component.

#### 3.1.3. Brightness Enhancement Network Based on CBAM

**Figure 5:**
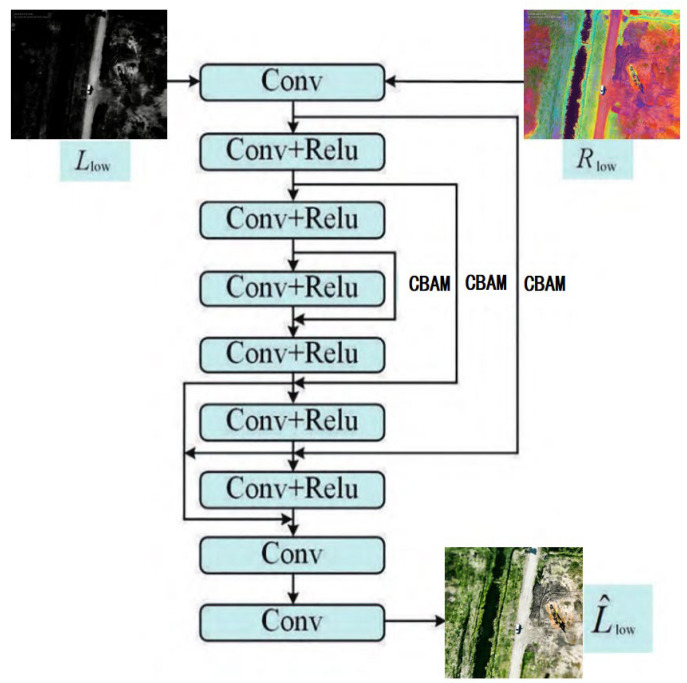
Brightness enhancement network architecture.

To obtain more detailed information, skip connections and CBAM are introduced into the brightness enhancement network to process the illumination map. Drawing inspiration from the U-Net architecture, the constructed brightness enhancement module is illustrated in Figure 5.

**Figure 6:**
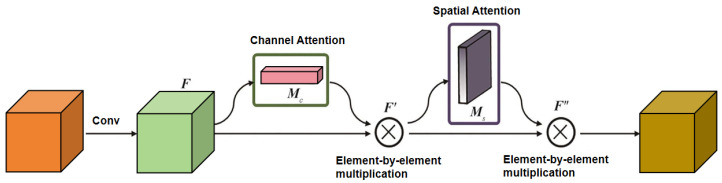
CBAM attention mechanism architecture.

In Figure 5, the brightness enhancement module consists of nine convolutional layers. One convolutional layer and five 3 × 3 Conv+ReLU layers form a U-Net architecture. Jump connections are introduced to link downsampling blocks with their corresponding mirrored upsampling blocks via element-wise addition, thereby forcing the network to learn residuals and preserve more details and semantic information. CBAM (convolutional block attention module) is embedded within the skip connections. As a representative hybrid attention mechanism [24], CBAM achieves adaptive focus on critical information across different dimensions by concatenating channel attention and spatial attention models. Subsequently, two 1 × 1 convolutions and sigmoid activation functions are employed to construct a complete channel attention map. In the default setting of this study, CBAM is embedded in the skip connections between the encoder and decoder, so as to adaptively emphasize leakage-related channels and spatial regions during cross-level feature transmission. The CBAM architecture is illustrated in Figure 6.

In Figure 6, the channel attention module Mc enhances feature extraction by assigning differential weights based on the informational importance of different feature channels. This enables the model to learn correlations among feature channels, thereby extracting effective information with greater precision. Simultaneously, it learns feature representations specific to certain channels in low-light scenarios, enhancing the effectiveness of illumination map augmentation. The subsequent spatial attention module Ms sets weights based on the spatial location information of features, enabling the model to learn dependencies across spatial dimensions of features. This further focuses on key regions within the image (such as the spatial distribution of oil slicks in oilfield spills) while suppressing interference from redundant background information. After the input features are convolved into feature F, it is first element-wise multiplied with the channel attention map Mc to yield feature F’. Feature F’ is then element-wise multiplied with the spatial attention map Ms to produce the optimized feature F″. The features processed by this module are fed into subsequent 3 × 3 Conv + ReLU layers and two additional 3 × 3 convolutional layers. The network employs a multi-scale fusion approach, concatenating feature outputs from each layer to minimize information loss, ultimately supporting the output of enhanced illumination components.

### 3.2. Image Recognition Algorithm Optimization

The FPN integrates deep semantic features from low-resolution, large receptive fields with shallow-level detail features from high-resolution, small receptive fields through a top-down feature propagation pathway. Theoretically, it can adapt to multi-scale detection requirements ranging from minute oil leaks to large-area oil films. However, FPN exhibits significant limitations in UAV-based oil leak detection: on one hand, its fixed receptive field size struggles to capture both the global distribution features of large oil films and the fine-grained textures of minute leaks, while also failing to accommodate cross-band information fusion requirements; on the other hand, features from different receptive fields are fused through simple element-wise addition, lacking effective interaction mechanisms. This results in insufficient coordination of multi-scale oil spill semantic information, making it difficult to distinguish oil spills from background interferences such as soil and vegetation.

To address the above issues, this paper proposes an attention-contextual feature pyramid network (AC-FPN), which is composed of a contextual enhancement module (CEM) followed by an attention module (AM). The overall structure can be expressed as AC-FPN = CEM + AM(CxAM + CnAM).

The purpose of CEM is to enhance the capability of the conventional FPN to model multi-scale contextual information in UAV-based oil leakage detection. Specifically, let the multi-level features generated by the backbone be denoted as {P3, P4, P5}. First, a 1 × 1 convolution is applied to each level to perform channel alignment, mapping all features to the same dimension and reducing the semantic discrepancy across levels before fusion. Based on the aligned features, each level is processed by a multi-branch receptive-field extraction unit, where parallel convolution operations are used to capture contextual responses at different scales. The small-receptive-field branch preserves the edges and fine-grained textures of minute leakage spots, the medium-receptive-field branch models the structural variations in strip-like seepage traces and local diffuse regions, and the large-receptive-field branch captures the spatial continuity of large-area diffuse oil films as well as their contextual relationships with surrounding soil, vegetation shadows, and equipment regions. The outputs of different receptive-field branches are concatenated along the channel dimension and further compressed and fused by convolution layers to generate the enhanced feature of each level.

To improve cross-scale interaction, CEM further introduces a dense inter-layer connection mechanism between adjacent pyramid levels. The enhanced feature at the current level receives not only top-down semantic information from the higher level, but also contextual information from neighboring levels, thereby shortening the propagation path of multi-scale features and strengthening the collaboration between shallow details and deep semantics. After multi-level contextual enhancement and cross-layer fusion, CEM outputs context-aware feature maps, which are then fed into the subsequent AM for redundant-background suppression and key-region emphasis.

**Figure 7:**
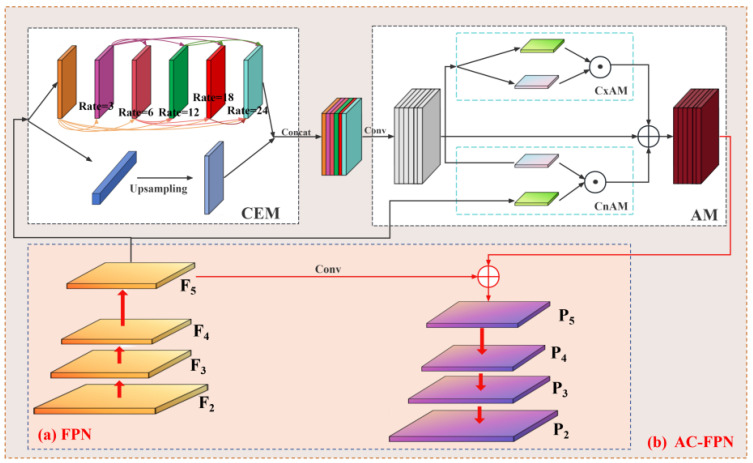
Comparison of AC-FPN and FPN Structures ((**a**): FPN, (**b**): AC-FPN).

It should be emphasized that the role of CEM is not merely to enlarge the receptive field of a single-level feature. Instead, it constructs a leakage-oriented contextual representation through three steps: channel alignment, multi-scale receptive-field extraction, and cross-level dense fusion. On the one hand, this design improves the joint modeling of minute leakage spots, strip-like seepage traces, and large-area diffuse oil films. On the other hand, it also provides richer but potentially redundant candidate features for the subsequent AM, which motivates the use of attention-based filtering. The architectural comparison between AC-FPN and traditional FPN is shown in Figure 7.

For the input feature *P_i_* at the *i*-th level, channel alignment is first performed by a 1 × 1 convolution:

(1) $${\hat{P}}_{i}\;=\;Conv_{1\times 1}(P_{i})$$

Then, ${\hat{P}}_{i}$ is fed into parallel multi-receptive-field branches to obtain contextual features at different scales:

(2) $$F_{i}^{\left(1\right)},F_{i}^{\left(2\right)},F_{i}^{\left(3\right)}\;=\;\Phi ({\hat{P}}_{i})$$

where Φ(·) denotes the parallel contextual extraction operation. The branch outputs are concatenated and fused by convolution to generate the enhanced feature of the current level:

(3) $$E_{i}\;=\;\mathrm{Conv}([F_{i}^{\left(1\right)};F_{i}^{\left(2\right)};F_{i}^{\left(3\right)}])$$

Finally, contextual features from adjacent levels are integrated to form the CEM output:

(4) $$C_{i}\;=\;\mathrm{Fusion}(E_{i-1},E_{i},E_{i+1})$$

where Fusion(·) denotes the dense cross-level connection and feature fusion operation. The output feature *C_i_* is then fed into the AM for attention-based filtering.

## 4. Experimental Results and Analysis

### 4.1. Datasets and Experimental Settings

In this study, two data usages should be distinguished clearly. The full UAV oilfield dataset contains 8629 images, which are divided into training, validation, and test sets with 6471, 548, and 1610 images, respectively, and is mainly used for the detection task. In contrast, the term “low-light paired dataset” refers to a subset used for low-light image enhancement training and evaluation, rather than to the entire 8629-image dataset. Specifically, each pair in the low-light paired subset consists of a low-light UAV oilfield image *I_L_* and a corresponding normal-light reference image *I_H_* from the same or highly similar inspection scene. These pairs are used in the enhancement stage, where both *I_L_* and *I_H_* are simultaneously input during training, while only *I_L_* is used during testing. To construct the paired subset, image pairs were selected from UAV acquisitions with consistent scene content and similar viewpoints under different illumination/exposure conditions. The paired images were manually screened to ensure semantic correspondence, and slight spatial deviations were corrected through basic alignment preprocessing before training. Therefore, the paired subset provides supervision for the enhancement network, while the full annotated dataset is used for the downstream detection task. It should be emphasized that paired supervision is not assumed to be available for all 8629 images. Only the subset with valid low-light/normal-light correspondence is used for enhancement training and visual comparison, whereas the detection stage is conducted on the larger annotated UAV dataset.

To avoid ambiguity, the detection label taxonomy is explicitly defined here. The detector is trained and evaluated as a four-class object detection task. These four classes are: grassland oil spill, flat-terrain oil spill, vehicle, and building shadow. Among them, grassland oil spill and flat-terrain oil spill are the two positive leakage categories, which distinguish leakage targets under different ground-background conditions. In contrast, vehicle and building shadow are hard-negative interference categories rather than leakage scenarios. They are explicitly annotated because, under UAV viewing angles and complex illumination, they frequently exhibit appearance patterns similar to oil leakage regions, such as dark irregular areas, elongated shadows, or local low-reflectance patches, and may therefore cause false alarms if treated only as unlabeled background. All categories are annotated using bounding boxes. This label design enables the detector not only to localize leakage targets, but also to learn explicit discrimination against typical confusing objects in oilfield scenes.

For evaluation, the original test split contains 1610 images. Among them, 1000 representative and challenging images covering nearly all major scene conditions were selected for detailed comparative analysis and visualization. These 1000 images follow the same four-class annotation protocol described above. This distinction is now clarified to avoid confusion between the original dataset split and the manually selected evaluation subset.

To improve reproducibility, the implementation details of both the low-light enhancement stage and the detection stage are further summarized as follows. All experiments were conducted on a workstation equipped with an AMD Ryzen 7 4800H CPU, Radeon Graphics 2.90 GHz, 32 GB RAM, and an NVIDIA GeForce RTX 2060 GPU. For the low-light enhancement stage, the improved RetinexNet was trained on the low-light paired subset using the Adam optimizer, with an initial learning rate of 1 × 10^−4^, a weight decay of 1 × 10^−5^, and a cosine decay learning-rate schedule. The batch size was set to eight, and the enhancement network was trained for 100 epochs. During training, the paired inputs were resized to 256 × 256, and only light augmentation operations that preserve pairwise correspondence were applied, including random cropping, horizontal flipping, and vertical flipping. For the detection stage, the enhanced images were fed into the improved YOLOv11 baseline detector, in which only the original FPN neck was replaced by the proposed AC-FPN, while the backbone and detection head remained unchanged. The detector was trained using SGD with an initial learning rate of 0.01, momentum of 0.9, a weight decay of 5 × 10^−4^, and a cosine annealing schedule. The batch size was set to eight, and the detector was trained for 100 epochs. The input image size was 640 × 640. During training, standard YOLO-style data augmentation was adopted, including Mosaic augmentation, random horizontal flipping, HSV color jittering, scaling, and translation. During inference, the confidence threshold was set to 0.25 and the IoU threshold for non-maximum suppression (NMS) was set to 0.70. Unless otherwise specified, mAP was reported at IoU = 0.50. The evaluation metrics include precision (P), recall (R), mAP, false alarm rate (FAR), and missed alarm rate (MAR).

### 4.2. Comparative Test

**Figure 8:**
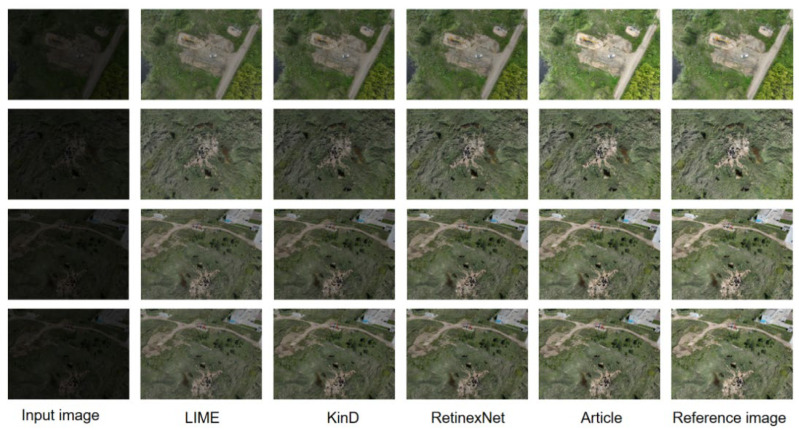
Visualization results of different low-light image enhancement methods.

Among the 1610 images in the original test split, 1000 representative challenging images covering nearly all major scene conditions were selected for detailed comparative evaluation and visualization. These images were annotated according to the same four-class taxonomy, including two leakage classes (grassland oil spill and flat-terrain oil spill) and two hard-negative interference classes (vehicle and building shadow). To validate the feasibility of the improved method, it was compared against other low-light image enhancement techniques on a low-light paired dataset. The effectiveness of the proposed method was validated by testing 15 low-light images, with four representative images selected for detailed comparative analysis, as shown in Figure 8.

As shown in Figure 8, images processed by the LIME method generally exhibit a darker background. While this enhances the visibility of shadow details to some extent, the method neglects the balance between contrast and clarity when addressing illumination issues. Consequently, contrast levels are reduced, resulting in an overall blurred appearance. Images processed by the KinD method exhibit improved contrast but suffer from insufficient overall brightness and noticeable distortion. This occurs because KinD excessively boosts brightness in certain areas during dynamic range adjustment, causing unnatural changes in color and structure. The RetinexNet method enhances both brightness and contrast but introduces noise in fine details, compromising overall image quality. The proposed method in this paper achieves visual results closer to the reference image in both overall background and fine details. It demonstrates superior performance in clarity and color enhancement within low-light regions. In summary, when processing low-light images, the proposed method not only effectively boosts brightness and contrast but also preserves image details and sharpness well, resulting in processed images that visually resemble reference images captured under normal lighting conditions.

Following this, the images captured by the drone were directly input into the improved YOLOv11 detector. This paper employs five commonly used technical metrics to reasonably evaluate different aspects of the model’s performance, namely: precision (P), recall (R), mean average precision (mAP), false alarm rate (FAR), and missed alarm rate (MAR). Precision measures the accuracy of the model in predicting positive samples, recall measures the model’s ability to correctly detect positive samples, and mean average precision (mAP) comprehensively evaluates the average accuracy of the model in predicting multiple categories. The detailed calculation formulas for these metrics are as follows:

(5) $$P\;=\;\frac{\mathrm{TP}}{\mathrm{TP}+\mathrm{FP}}$$

(6) $$R=\frac{\mathrm{TP}}{\mathrm{TP}+\mathrm{FN}}$$

where *P* is precision, *R* is recall, *TP* is the number of correctly predicted positive samples, *FP* is the number of incorrectly predicted positive samples, and *FN* is the number of positive samples not predicted.

(7) $$\mathrm{mAP}=\frac{1}{N}\sum_{i=0}^{N}\int_{0}^{1}P_{i}(R)\mathrm{dR}$$

**Figure 9:**
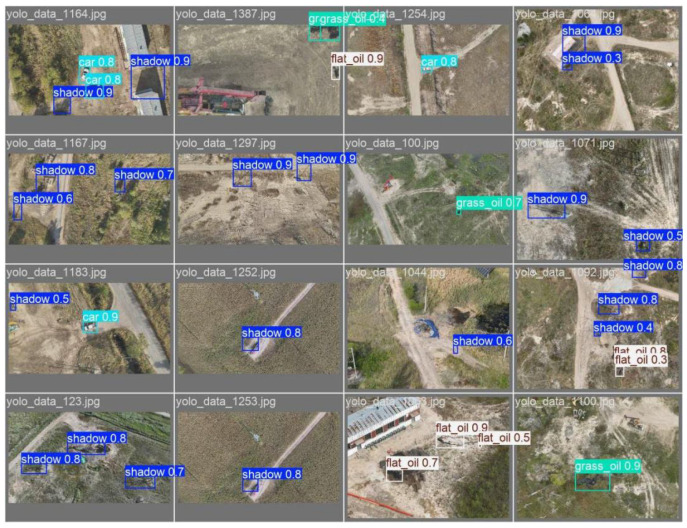
Visualization of the recognition method after low-light image enhancement.

In the formula, *N* denotes the number of sample categories, *P_i_* represents the accuracy rate of category *i*, and *R* denotes the recall rate. Partial example results of the detection are shown in Figure 9, with the data results presented in Table 1.

**Figure 10:**
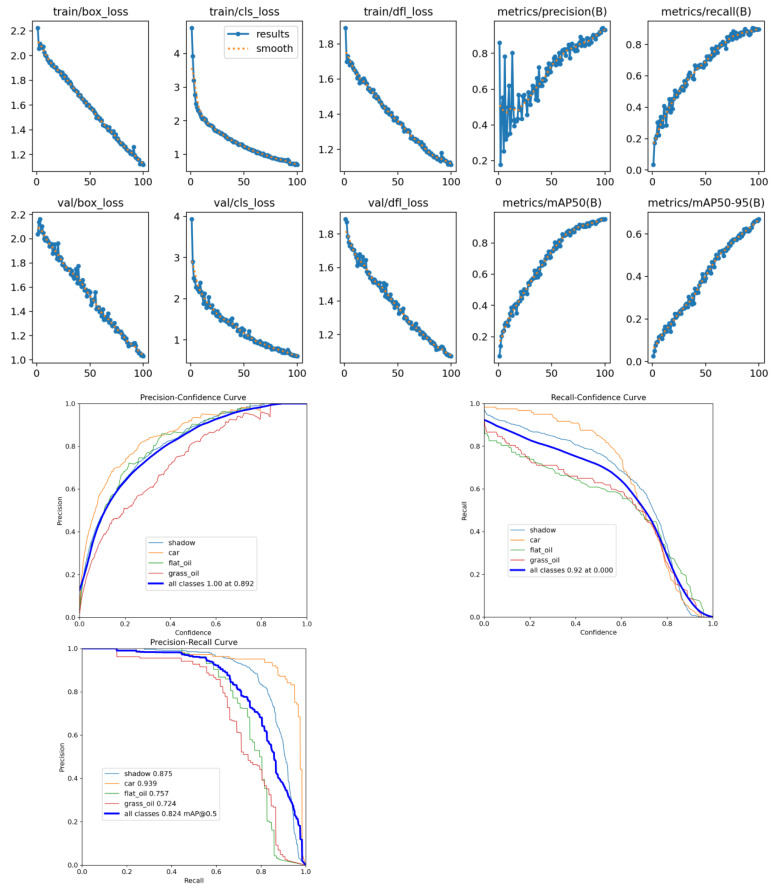
Key loss curves and performance evaluation curves during model training.

To provide a more comprehensive and intuitive representation of the model training process’s stability and the detailed characteristics of its detection performance, Figure 10 displays key loss variation curves and performance evaluation curves during the model training phase. The upper section of the figure corresponds to the bounding box loss (box_loss), classification loss (cls_loss), and distribution focus loss (dfl_loss) for both the training and validation sets, alongside real-time monitoring of precision and recall during training. The trends reveal that all loss metrics steadily converge and stabilize as training epochs increase, without noticeable oscillations or overfitting. This demonstrates the robust training stability and model generalization capability of the designed “low-light enhancement-accurate detection” collaborative architecture. The lower section includes precision-confidence curves and recall-confidence curves, and precision-recall (P-R) curve. These further quantify the model’s performance across different confidence thresholds, not only intuitively reflecting its adaptability to diverse target categories but also validating the synergistic effect between the low-light enhancement module and the AC-FPN feature fusion module. This provides visual support for the precise detection of multi-scale oil spills under complex lighting conditions.

### 4.3. Ablation Experiment

#### 4.3.1. Overall Module Ablation

To validate the effectiveness of each core innovative module in the proposed “Low-Light Enhancement-Precision Detection” collaborative optimization method, comparative experiments were conducted by sequentially removing or replacing key components. Quantitative analysis was performed on the multi-scale feature aggregation module, NAFNet denoising module, CBAM attention module, and AC-FPN (CEM + AM) module to detection performance, clarifying their respective mechanisms in complex lighting adaptation, noise suppression, detail preservation, and multi-scale object detection. Experimental results are presented in Table 2.

The ablation study results reveal that using “RetinexNet + YOLOv11 (without additional modules)” as the baseline group (corresponding to Group 1 in the table), introducing a single optimization module individually yields varying degrees of improvement in object detection performance: Adding only the multi-scale aggregation module increased the mean average precision (mAP) from 79.67% to 82.34%, while simultaneously reducing the false alarm rate (FAR) and missed alarm rate (MAR). This indicates that the multi-scale aggregation module effectively preserves the fine details of small oil films under complex lighting conditions, thereby lowering the risk of missed detections. When only the NAF-Net module was introduced, mAP increased to 81.56%, demonstrating its ability to suppress noise in low-light images and reduce the impact of interfering features on detection. Integrating only the AC-FPN module raised mAP to 83.15%, highlighting its fundamental effectiveness in multi-scale oil leak feature fusion and redundant interference suppression. However, when different optimization modules are combined, the performance improvement significantly exceeds the independent effects of individual modules: For example, the “Multi-scale Aggregation + NAF-Net” combination (Group 4) further increased mAP to 84.18%; the synergistic collaboration of enhanced submodules (“Multi-scale Aggregation + NAF-Net + CBAM,” Group 7) achieved an mAP of 85.72%, with false positive and false negative rates reduced by approximately 1 percentage point compared to the baseline group, validating the synergistic enhancement logic among low-light enhancement submodules.

Finally, when fully integrating the complete low-light enhancement module (multi-scale aggregation + NAF-Net + CBAM) with the detection module AC-FPN (corresponding to Group 9 in the table), the precision (P) of object detection reached 94.25%, with the mean average precision (mAP) rising to 87.54%. The false alarm rate (FAR) and missed detection rate (MAR) decreased to 0.98% and 1.02%, respectively—compared to the baseline group, mAP improved by 7.87 percentage points, while FAR and MAR decreased by over 2 percentage points. This demonstrates that both proposed strategies—the “low-light enhancement submodule optimization” and the “feature fusion optimization for the detection network”—play crucial roles in improving oil spill detection accuracy under complex lighting conditions in oilfields. Moreover, their synergistic integration maximizes the combined benefits of detail preservation, noise suppression, and feature fusion, achieving a significant leap in detection performance.

#### 4.3.2. Ablation on CBAM Insertion Positions

To further determine the optimal deployment strategy of CBAM in the brightness enhancement network, an additional ablation study on attention insertion positions was conducted. Under the condition that all other network structures and training settings remain unchanged, three comparison settings were evaluated:

(A) CBAM embedded only in the skip connections;
(B) CBAM embedded only before each convolutional layer in the decoder;
(C) CBAM embedded in both the skip connections and before each convolutional layer in the decoder.

All settings were trained and evaluated on the same training and testing sets. The detection performance was quantitatively compared using precision (P), recall (R), mAP, false alarm rate (FAR), and missed alarm rate (MAR).

The results are presented in Table 3. It can be observed that embedding CBAM only in the skip connections achieves the best overall performance. This indicates that the skip-connection pathway is a more suitable position for attention modulation, because it directly regulates the cross-level transmission of shallow detail features and deep semantic information, which is particularly beneficial for preserving the weak boundaries and fine textures of minute oil leakage targets under complex illumination. When CBAM is embedded only before each convolutional layer in the decoder, the model improves feature refinement during the reconstruction stage, but lacks sufficient attention guidance during cross-layer feature transmission, resulting in relatively lower detection performance. When CBAM is embedded in both locations, the model does not obtain further improvement; instead, a slight performance drop is observed. This suggests that excessive attention insertion may introduce redundant feature reweighting, and suppress useful low-level responses.

Therefore, embedding CBAM in the skip connections is adopted as the final configuration in this paper, as it provides the best balance between detection accuracy, robustness, and model efficiency.

As shown in Table 3, Setting A, where CBAM is embedded only in the skip connections, achieves the highest P, R, and mAP, while also obtaining the lowest FAR and MAR. Compared with Setting B, the mAP is improved by 0.86 percentage points, indicating that attention modulation during cross-level feature transmission is more effective than applying attention only in the decoder reconstruction stage. Compared with Setting C, although dual-position embedding introduces more attention operations, it does not further improve the detection accuracy; instead, the mAP decreases by 0.33 percentage points. This demonstrates that excessive attention insertion may lead to redundant feature suppression. Therefore, the skip-connection embedding strategy is retained in the final model.

### 4.4. Practical Deployment Discussion for UAV/UAM Systems

Beyond algorithmic accuracy, the practical deployment of aerial inspection systems depends on platform endurance, payload budget, onboard computing resources, and air–ground communication reliability. Although the present study is primarily designed for low-altitude UAV-based oilfield inspection rather than passenger-oriented urban air mobility (UAM), the deployment considerations are relevant to a broader class of intelligent aerial systems. Recent UAM research has shown that payload capacity and flight endurance remain critical bottlenecks for aerial platforms, and that energy-system design must be evaluated jointly with mission requirements and power-to-weight constraints. In particular, hybrid power system studies for eVTOL/UAM vehicles emphasize that endurance improvement is inseparable from payload and power-density trade-offs [25].

From this perspective, the practical value of the proposed framework lies in its deployment-oriented design. On the one hand, the improved YOLOv11 detector preserves the lightweight characteristics of the original architecture while replacing only the neck with AC-FPN, which is beneficial for edge-side inference under limited onboard resources. On the other hand, the collaborative “enhancement-detection” pipeline is particularly suitable for missions in which image quality is strongly affected by illumination variation, such as dawn, dusk, backlighting, or shadow-interfered inspections in large oilfield areas. These properties make the framework applicable not only to routine UAV patrols in oilfields, but also to future aerial inspection platforms that require a balance among perception accuracy, endurance, and onboard computational efficiency.

In addition, practical aerial deployment increasingly relies on air–ground collaborative communication. A recent study on air-to-ground integrated mobile ad hoc networks highlighted that dynamic topology, limited node resources, and unstable communication opportunities are key challenges in aerial mission systems, and proposed routing strategies based on position prediction and resource-aware relay selection. This is relevant to the present work because real oilfield inspection systems often require image transmission, alarm reporting, and task coordination between UAVs and ground stations. Therefore, the proposed detection framework can be viewed as the perception layer of a larger intelligent aerial inspection system, where future work may integrate edge inference, event-triggered transmission, and air–ground collaborative scheduling to support more complex UAV/UAM application scenarios [26].

Overall, the proposed method is currently validated on UAV oilfield inspection tasks, but its engineering implications extend to broader aerial mobility systems: a practically useful platform should not only achieve accurate visual detection under complex illumination, but also remain compatible with constraints in endurance, payload, communication, and onboard intelligence.

## 5. Summary

This paper addresses the core engineering challenges in drone inspections of onshore oilfields, where complex lighting conditions lead to low accuracy and insufficient robustness in identifying oil leaks. We systematically investigate intelligent oil-leak-detection technology based on enhancement mechanisms. First, we establish a collaborative technical framework integrating “low-light image enhancement with multi-scale precise target detection,” overcoming the adaptability limitations of traditional single detection models in complex lighting scenarios. This marks the first implementation of an illumination-adaptive oil-leak-detection solution tailored for onshore oilfield inspection scenarios, addressing dual research gaps: insufficient specificity of existing visual detection technologies for onshore oilfields and poor compatibility between low-light image enhancement (LLIE) methods and oil-leak-detection tasks. Second, an enhanced RetinexNet low-light enhancement module was proposed. This innovation organically integrates a multi-scale feature aggregation-decomposition mechanism, NAFNet denoising network, and CBAM attention mechanism. It simultaneously achieves synergistic optimization of oil spill detail preservation and noise suppression while effectively boosting image brightness, resolving the technical challenges of overexposure and detail loss common in traditional enhancement methods. Third, we optimized the YOLOv11 detection model by introducing an AC-FPN feature fusion module. This enhances feature extraction and interaction capabilities for multi-scale oil spill targets (especially minute spill points), significantly improving the model’s recognition accuracy and interference resistance in complex backgrounds. Extensive comparative experiments, ablation studies, and visual validation demonstrate that the proposed method achieves 94.25% precision and 87.54% mAP on the test set. Its overall performance significantly outperforms existing LLIE methods and mainstream object detection models, demonstrating stable adaptability to the practical demands of complex lighting conditions in drone-based edge-side inspection scenarios. This research not only establishes an efficient and reliable technical paradigm for identifying oil leakage points in complex lighting conditions within oilfields but also provides valuable insights for detecting small objects in low-quality images across industrial scenarios. It holds significant engineering and academic value for advancing the intelligent and efficient upgrading of oilfield safety production monitoring systems. Future work will further investigate the integration of the proposed perception framework with energy-constrained aerial platforms and air–ground collaborative communication mechanisms, so as to support practical deployment in broader UAV/UAM inspection systems.

## Figures and Tables

**Table 1:** Identification results data.

	P	R	MAP	FAR	MAR
Augmented pre-training dataset	87.21%	75.43%	79.67%	3.24%	3.56%
Augmented Dataset	93.75%	82.35%	87.54%	0.98%	1.02%

**Table 2:** Ablation experiment results.

RetinexNet	YOLOv11	Multi-Scale Aggregation	NAF-Net	CBAM	AC- FPN	P%	R%	MAP%	FAR%	MAR%
√	√					87.21	75.43	79.67	3.24	3.56
√	√	√				89.15	78.26	82.34	2.87	3.12
√	√		√			88.32	77.14	81.56	2.98	3.25
√	√	√	√			90.47	80.12	84.18	2.53	2.81
√	√	√		√		89.83	79.35	83.27	2.72	2.96
√	√		√	√		89.26	78.58	82.75	2.81	3.04
√	√	√	√	√		91.65	81.43	85.72	2.21	2.54
√	√				√	89.74	79.21	83.15	2.75	3.01
√	√	√	√	√	√	94.25	82.35	87.54	0.98	1.02

**Table 3:** Ablation results of different CBAM insertion positions.

Setting	Skip Connection	Before Each Decoder Conv	P%	R%	MAP%	FAR%	MAR%
A	√		94.25	82.35	87.54	0.98	1.02
B		√	93.41	81.27	86.68	1.17	1.21
C	√	√	93.96	81.94	87.21	1.05	1.10

## Data Availability

No new data were created or analyzed in this study.
